# Supplementary material for: Protocol for the COG-UK hospital-onset COVID-19 infection (HOCI) multicentre interventional clinical study: evaluating the efficacy of rapid genome sequencing of SARS-CoV-2 in limiting the spread of COVID-19 in UK NHS hospitals
Source: BMJ Open. 2022 Apr 18;12(4):e052514. doi: 10.1136/bmjopen-2021-052514 (PMC9019828; doi:10.1136/bmjopen-2021-052514)
Supplement: Author's manuscript [file bmjopen-2021-052514.draft_revisions.pdf]

BMJ Open is committed to open peer review. As part of this commitment we make the peer review history of every article we publish publicly available.

When an article is published we post the peer reviewers' comments and the authors' responses online. We also post the versions of the paper that were used during peer review. These are the versions that the peer review comments apply to.

The versions of the paper that follow are the versions that were submitted during the peer review process. They are not the versions of record or the final published versions. They should not be cited or distributed as the published version of this manuscript.

BMJ Open is an open access journal and the full, final, typeset and author-corrected version of record of the manuscript is available on our site with no access controls, subscription charges or pay-per-view fees (http://bmjopen.bmj.com).

If you have any questions on BMJ Open's open peer review process please email info.bmjopen@bmj.com

BMJ Open

Protocol for the COG-UK hospital onset COVID-19 infection (HOCI) multicentre interventional clinical study: evaluating the efficacy of rapid genome sequencing of SARS-CoV-2 in limiting the spread of COVID-19 in United Kingdom NHS hospitals

Journal:	*BMJ Open*
Manuscript ID	bmjopen-2021-052514
Article Type:	Protocol
Date Submitted by the Author:	17-Apr-2021

Complete List of Authors:	Blackstone, James; University College London, Comprehensive Clinical Trials Unit<br>Stirrup , Oliver ; University College London, Institute for Global Health<br>Mapp, Fiona; UCL, Institute for Global Health<br>Panca, Monica; University College London, Comprehensive Clinical Trials Unit<br>Copas, Andrew; University College London, Institute for Global Health<br>Flowers, Paul; University of Strathclyde<br>Hockey, Leanne; UCL, Comprehensive Clinical Trials Unit<br>Price , James ; Imperial College London, Department of Infectious Disease<br>Partridge, David; Sheffield Teaching Hospitals NHS Foundation Trust, Department of Virology<br>Peters, Christine; NHS Greater Glasgow and Clyde<br>de Silva, Thushan; The University of Sheffield, Department of Infection, Immunity and Cardiovascular Disease<br>Nebbia, Gaia; Guy's and St Thomas' NHS Foundation Trust, Department of Infectious Diseases<br>Snell, Luke; Guy's and St Thomas' NHS Foundation Trust, Department of Infectious Diseases<br>McComish, Rachel; University College London, Comprehensive Clinical Trials Unit<br>Breuer, Judith; UCL

Keywords:	COVID-19, Infection control < INFECTIOUS DISEASES, MOLECULAR BIOLOGY, Epidemiology < INFECTIOUS DISEASES

SCHOLARONE™
Manuscripts

Protocol for the COG-UK hospital onset COVID-19 infection (HOCI) multicentre interventional clinical study: evaluating the efficacy of rapid genome sequencing of SARS-CoV-2 in limiting the spread of COVID-19 in United Kingdom NHS hospitals

Authors: James Blackstone[1]; Oliver Stirrup[2]; Fiona Mapp[2]; Monica Panca[1]; Andrew Copas[2]; Paul Flowers[3]; Leanne Hockey[1]; James Price[4]; David Partridge[5]; Christine Peters[6]; Thushan de Silva[7]; Gaia Nebbia[8]; Luke B. Snell[8]; Rachel McComish[1]; the COVID-19 Genomics UK (COG-UK) Consortium; Judith Breuer[9].

1 Comprehensive Clinical Trials Unit, Institute of Clinical Trials and Methodology, UCL, London, UK

2 Institute for Global Health, UCL, London, UK

3 School of Psychological Sciences and Health, University of Strathclyde, Glasgow, UK

4 Faculty of Medicine, Department of Infectious Disease, Imperial College London, UK

5 Sheffield Teaching Hospitals NHS Foundation Trust, Sheffield, UK

6 Queen Elizabeth University Hospital, Greater Glasgow and Clyde Heath Board, Glasgow, UK

7 Department of Infection, Immunity and Cardiovascular Disease, University of Sheffield, Sheffield, UK

8 Department of Infectious Diseases, Guy's and St Thomas' NHS Foundation Trust, London, UK

9 Infection, Immunity and Inflammation, Great Ormond Street Institute of Child Health, UCL, London, UK

Word count: 4,116

Corresponding Author:
Prof. Judith Breuer, j.breuer@ucl.ac.uk
Institute of Child Health, University College London, 30 Guilford Street, London, WC1N 1EH, UK

For peer review only

ABSTRACT

Introduction

Nosocomial transmission of severe acute respiratory syndrome coronavirus 2 (SARS-CoV-2) has been a significant cause of mortality in National Health Service (NHS) hospitals during the coronavirus disease 2019 (COVID-19) pandemic. This study aims to evaluate the impact of rapid whole genome sequencing of SARS-CoV-2, supported by a novel probabilistic reporting methodology, to inform infection prevention and control (IPC) practice within NHS hospital settings.

Methods and analysis

COG-UK HOCI (COG-UK Consortium Hospital-Onset COVID-19 Infections study) is a multicentre, prospective, interventional, superiority study. Eligible patients must be admitted to hospital with first confirmed SARS-CoV-2 PCR positive test result >48h from time of admission, where COVID-19 diagnosis not suspected upon admission. The projected sample size for 14 participating sites over winter-spring 2020/2021 in the United Kingdom is 2,380 patients. The intervention is the return of a sequence report, within 48 hours in one phase (rapid local lab) and within 5-10 days in a second phase (mimicking central lab), comparing the viral genome from an eligible study participant with others within and outside the hospital site. The primary outcomes are incidence of Public Health England (PHE)/IPC-defined SARS-CoV-2 hospital-acquired infection during the baseline and two interventional phases, and proportion of hospital-onset cases with genomic evidence of transmission linkage following implementation of the intervention where such linkage was not suspected by initial IPC investigation. Secondary outcomes include incidence of hospital outbreaks, with and without sequencing data; actual and desirable changes to IPC actions; periods of healthcare worker (HCW) absence. Health economic analysis will be conducted to determine cost-benefit of the intervention. A process evaluation using qualitative interviews with HCWs will be conducted alongside the study.

Ethics

The protocol has been approved by the National Research Ethics Service Committee (Cambridge South 20/EE/0118). This manuscript is based on protocol version 5.0.

Study Registration number

ISRCTN50212645

For peer review only

Strengths and limitations of this study

- The COG-UK HOCI study harnesses the infrastructure of the UK's existing national COVID-19 genome sequencing platform to evaluate the specific benefit of sequencing to hospital infection control.
- The evaluation is thought to be the first interventional study globally to assess effectiveness of genomic sequencing for infection control in an unbiased patient selection in secondary care settings.
- The findings are likely to have wider applicability in future decisions to utilise genome sequencing for infection control of other pathogens (such as influenza, respiratory syncytial virus, norovirus, clostridium difficile and antimicrobial resistant pathogens) in secondary care settings.
- The study has been awarded UK NIHR Urgent Public Health status, ensuring prioritised access to NIHR Clinical Research Network (CRN) research staff to recruit patients.
- The study does not have a randomised controlled design due to the logistics of managing this against diverse standard practice.

For peer review only

INTRODUCTION

Background

Hospitals are recognised to be a major risk for the spread of infections despite the universal introduction of infection control measures. For severe acute respiratory syndrome coronavirus 2 (SARS-CoV-2), nosocomial spread of infection presents an additional and significant health risk to patients and healthcare workers (HCW). During epidemics, infection prevention and control (IPC) practice is further complicated by the difficulties of distinguishing community and hospital-acquired infections. This can lead to erroneous identification of nosocomial transmission leading to unnecessary IPC efforts. True nosocomial transmission events may be missed with appropriate interventions not performed, thereby putting patients and HCW at increased risk. The epidemiological determination of infection timing for SARS-CoV-2 is made especially challenging by its prolonged incubation period in distinguishing community from nosocomial transmission.

There is now good evidence that genome sequencing of epidemic viruses, together with standard IPC, better defines nosocomial transmissions and, depending on the virus, better identifies routes of transmission, than IPC alone.[1-3] To date, all studies have been retrospective. However, the development of rapid sequencing methods enable sequencing of potentially linked or unlinked SARS-CoV-2 genomes within 48 hours. This timescale is short enough to inform clinically relevant IPC decisions in near-real-time.

While SARS-CoV-2 has a low mutation rate (estimated at around 2.5 changes per genome per month), sufficient viral diversity exists to identify cases where patient and HCW infections that are clustered in time and space are in fact due to different SARS-CoV-2 genotypes.[4] Such information could rapidly exclude nosocomial transmission as the cause of the cluster and redirect IPC intervention to where needed most.

Confirmation of SARS-CoV-2 transmission to patients and healthcare workers may be more challenging with a single observed mutation between two genomes, feasibly representing anything between one and ten transmissions. Identical genomes will not necessarily evidence a close link between two cases. Nonetheless, by comparing genotypes detected within the hospital setting and the surrounding community, it may be possible to reveal unsuspected nosocomial transmission where comparatively uncommon genotypes are apparently linked or cluster in time and space.

The COG-UK initiative aims to sequence as many SARS-CoV-2 viruses as possible across the UK for public health planning. It also provides an important and unique opportunity to test

For peer review only - http://bmjopen.bmj.com/site/about/guidelines.xhtml

whether viral sequence data produced in near-real-time could also reduce uncertainties around nosocomial transmission events, better direct IPC effort, improve hospital functioning and reduce the role of hospitals as a source of infection to the community.[5]

COG-UK HOCI* will harness the COG–UK sequencing platform, with its mixed model of smaller sequencing hubs located close to hospitals and a large centralised hub sequencing most viruses. It will identify not only whether rapid viral sequencing is useful for patient management, but how time-critical this might be; turnaround times for sequence data from a central hub are likely to be longer (5-10 days) than those from local sequencing hubs (<48 hours).

*Note that while 'hospital-onset COVID-19 infections' (HOCI) was the preferred term at the study's inception, evolution of the terminology now favours references be made to 'hospital-onset SARS-CoV-2 infections.'

Objective

The study will evaluate the contribution of whole-genome sequencing combined with a novel viral sequence report design to IPC investigation and response to cases of hospital onset coronavirus disease 2019 (COVID-19) infection, and whether this can reduce the overall incidence of hospital-acquired infections.[6]

METHODS AND ANALYSIS

Study design

COG-UK HOCI is a prospective, interventional, superiority clinical study, comprising three distinct phases with a possible fourth, dependant on interim data analysis.

In the first phase, all sites will collect baseline (non-interventional) eligible patient data for a period of four weeks to characterise each site's usual practice in infection control in response to hospital-onset COVID-19 cases. This phase may include standard of care use of genome sequencing (e.g. limited outbreak response analysis).

In the second and third phases, the study intervention will be applied on top of standard of care infection control practices.

The second phase requires 'rapid' turnaround of genome sequencing and sequence linkage

For peer review only

report generation (i.e. within 48 hours of first diagnostic SARS-CoV-2 polymerase chain reaction positive result). This phase will be applied to all hospital onset COVID-19 cases meeting the eligibility criteria over an 8 week period.

The third phase is similar to the second, except that a 5-10 day turnaround time of genome sequencing and sequence report generation should be applied to mimic the use of a central sequencing laboratory. This phase will apply to all sites and last for 4 weeks.

The second and third phases may be applied in the reverse order at some sites, both for logistical reasons (i.e. fine-tuning of rapid turnaround of whole-genome sequences) and also to ensure differences between sites in the calendar dates of each phase.

Upon review of interim analysis data, the study's joint oversight committee may recommend a fourth phase for all sites comprising a second baseline period; this would be applied where the initial baseline data collection period occurred at a time of very high or low COVID-19 prevalence at the sites, whereby collecting data on standard practice could be unviable.

This will be a sequential study, with each NHS Trust acting as its own control.

The total study duration per site will accordingly be 16-20 weeks, though it is likely that pauses in data collection will occur over the winter holiday break period due to most sequencing labs closing or moving to skeletal operations during this time.

INTERVENTION

Overview

The study intervention is a SARS-CoV-2 genomic sequencing data report (see Figure 1) delivered to the NHS site's Infection Prevention and Control (IPC) teams, either within 24-48 hours of the sample from the patient being confirmed as positive for SARS-CoV-2 (rapid genomic sequencing locally), or within 5-10 days (local genomic sequencing to mimic use of a centralised lab).[7]

Microbiology and IPC teams will be trained to interpret the results. An expert sequence interpretation team (a sub-set of the Study Team) will be available seven days a week by phone and online to discuss results where required with IPC teams, and to provide guidance on best practice.

For peer review only

Genomic sequence reporting tool

The genomic sequence report tool combines epidemiological and consensus sequence data in order to provide a rapid assessment of the probability of hospital acquired infection (HAI) among new HOCI study cases and to identify infections that could plausibly constitute a hospital outbreak event. [8]

The internal calculations use a combination of admission-to-symptom onset intervals and differences between the observed proportion of close sequence matches (defined as a maximum pairwise difference of 2 single nucleotide polymorphisms; SNPs) for viral samples obtained from various locations (i.e. same ward, same hospital, within the community) to estimate the probability that the patient's SARS-CoV-2 infection was acquired in hospital.

The report generation algorithm is designed to run quickly and reliably, without the need for local model checking, thereby reducing the need for expert bioinformatics input during operation.

Sequence reports

The summary report for each focus sequence submitted, corresponding to a single HOCI case, will comprise:

1. The lineage assignment for the focus sequence.
2. A list of the details of any close sequence matches from samples on the same ward as the focus sequence in the previous three weeks, with estimated probability of infection having occurred from a source on the ward (reported as low, moderately low, probable, high, very high).
3. A list of the details of any close sequence matches from samples obtained within the hospital but not same ward as the focus sequence in the previous three weeks, with estimated probability of infection having occurred from a source in the institution (reported as low, moderately low, probable, high, very high).
4. Estimated probability of infection from a visitor to the ward (reported as low, moderately low, probable, high, very high).
5. Estimated probability of community-acquired infection (reported as low, moderately low, probable, high, very high).
6. A graphical summary displaying sample dates of close sequence matches at the ward and hospital levels, along with the total number of samples obtained over the previous three weeks.

For peer review only - http://bmjopen.bmj.com/site/about/guidelines.xhtml

A detailed report will also be returned to virology labs for each focus sequence, containing additional details of all the recent sequences obtained within the given ward and hospital that have contributed to the output summarised in the summary report, and their similarity with respect to the focus sequence.

Allocation of intervention

All sites will engage in the various study phases sequentially; there will be no allocation of intervention either by site or at patient-level for this study.

POPULATION

Setting

Fourteen NHS Trusts/Heath Boards across England and Scotland will participate. Sites will be set up either as all hospitals within a Trust or a single hospital selected from within the Trust. This decision will be site-led and based on available research team, infection control team and sequencing resource. Sites will be selected to span tertiary referral centres through to district hospitals, primarily in urban or suburban settings.

Inclusion criteria

Patients will be considered eligible only where they are an inpatient with first confirmed positive test for SARS-CoV-2 >48 hours after admission, where they were not suspected to have COVID-19 at time of admission.

Participants may be of any age to be included in the study.

There are no exclusion criteria.

Recruitment

Viral sequencing will be attempted for every confirmed case of SARS-CoV-2 in hospital patients and HCW, but it is not possible to assess clinical and infection control outcomes for every confirmed case. This study will therefore focus on the subset of patients with hospital-onset SARS-CoV-2, since this is where the additional knowledge potentially provided by viral sequencing is likely to have the greatest impact for IPC teams.

For peer review only

Patient consent

Consent for participant (both patient and healthcare worker) involvement will not be sought for COG-UK HOCI study. This approach relies on the Health Service (Control of Patient Information) Regulations 2002 (SI 1438), specifically Regulation 3 (Communicable disease and other risks to public health), and Regulation 7 (the processing of confidential information for medical research). This approach was reviewed and approved by a Research Ethics Committee.

STUDY OUTCOMES

Primary outcomes

1. Incidence rate of PHE/IPC-defined SARS-CoV-2 HAIs (defined as SARS-CoV-2 cases with an interval of ≥8 days from admission to symptom onset, if known, or sample date), measured as incidence rate of recorded cases per week per 100 inpatients, during each phase of the study.
2. Identification of linkage to individuals within an outbreak of SARS-CoV-2 nosocomial transmission using sequencing report data for HOCIs in whom this was not identified by pre-sequencing IPC evaluation, for each enrolled patient during study phases in which the sequence reporting tool is in use.

Secondary outcomes

1. Incidence rate of IPC-defined SARS-CoV-2 hospital outbreaks, defined as cases of hospital transmission linked by location and with intervals between diagnoses no greater than 28 days, measured as incidence rate of outbreak events per week per 100 inpatients during each phase of the study.
2. Incidence rate of IPC+sequencing-defined SARS-CoV-2 hospital outbreaks involving HOCI cases, defined as for IPC-defined SARS-CoV-2 hospital outbreaks with the additional condition of clustering of viral sequences and measured as outbreak events per week per 100 inpatients during study phases in which the sequence reporting tool is in use. Genetic clusters are defined as having maximum viral sequence pairwise SNP distance of 2 between each individual included and their nearest neighbour within the cluster.
3. Changes to IPC actions implemented following receipt of SARS-CoV-2 sequence report, for each enrolled patient during study phases in which the sequence reporting tool is in use.

For peer review only

4. Changes to IPC actions that would ideally have been implemented but may not have been following receipt of SARS-CoV-2 sequence report, for each enrolled patient during study phases in which the sequence reporting tool is in use.
5. Health economic benefit of both slow and rapid sequencing reports to IPC against baseline.
6. The number of HCW periods of sickness/self-isolation, assessed as a proportion of the number of staff usually on those wards impacted by HOCI cases, for all phases of the study.

Exploratory outcomes

Additionally, descriptive summaries of sequence report results will be generated, including number of close sequence matches on ward and within hospital; probability of infection source; whether healthcare workers are reported within close sequence matches.

For the process evaluation, the qualitative team will seek to understand how the intervention worked in practice across a representative sample of study sites (n=5). This will include how the context shaped the intervention; how key intervention components and causal mechanisms operated for IPC teams and hospital planners, and how the intervention changed the study outcomes.

SAMPLE SIZE AND POWER

The projected total sample size is 2,380 patients.

There is uncertainty in the number of HOCIs that will be identified at each site during each of the intervention periods, with the rapid testing phase being eight weeks' duration. Based on clinical experience of first wave and discussion with the principal investigators, we assume there may be an average of 10 HOCIs/week per site during this intervention period, a total of 80 per site. Within a typical site this will allow us to estimate the proportion of HOCIs with genotypic linkage to any other case(s) not detected by IPC processes with minimum precision of +/- 9.4%. Similarly, we can estimate the proportion of HOCIs where an action is taken that would not have occurred without sequencing within +/- 9.4%. We shall also calculate pooled estimates of these proportions across the 14 sites, leading to estimation within +/- 6.5% assuming an intracluster correlation coefficient of 0.05.

For peer review only - http://bmjopen.bmj.com/site/about/guidelines.xhtml

Comparing the proportion of HOCIs with genotypic linkage to any other case(s) not detected by IPC processes between rapid testing and slow testing phases across all sites, the study would have at least 80% power to detect a percentage point difference of 11%. This corresponds to a two-sided test with alpha=0.05, considering proportions of 55.5% vs 44.5% which would be associated with minimum power for a difference of this magnitude.

For the outcome of weekly incidence of IPC-defined HOCIs, using an approximate Normal distribution for weekly counts there is 86.7% power to demonstrate a reduction from 12 IPC-defined HOCIs per week in the baseline phase to 10 per week during the rapid testing phase across all sites, under 5% significance level two-tailed testing. However, these calculations correspond to a variance of 12 for weekly counts based on the Poisson distribution, but the presence of over-dispersion of weekly counts would lead to a lower power to detect a difference. Using an overdispersion parameter of 0.82 based on retrospective analysis of data from Sheffield and Glasgow (dataset as described by Stirrup et al.) results in 81% power to detect a reduction in mean weekly incidence from 12.5 to 10.[6]

DATA MANAGEMENT AND PROTECTION

All study documentation at site will be held in restricted access areas and stored securely by study team members. Data will be entered by sites into a secure, validated online database (Elsevier MACRO v4) and accessible only by delegated team members of that site, and by delegated staff from the coordinating centre.

Case Report Forms (CRFs) for the study will identify patients using a unique five-digit study identifier, year of birth, and initials. Under the Data Protection Act 2018, the latter identifiers will be considered 'personally identifying' and will be treated as such by both the site team and coordinating centre team.

Where written communication (e.g. data queries) on individual patient cases is necessitated between sites and the coordinating centre, only the study identifier should be used in the first instance.

Any transfer of documentation containing personally identifying data between site and coordinating centre will be subject to AES-256 industry-standard encryption.

For peer review only

STATISTICAL ANALYSIS

Summary statistics will be presented for each study phase (baseline, rapid local lab, and central lab interventions) and each site, which will be percentages for binary outcomes such as whether transmission linkage for each HOCI was previously undetected. The frequency of IPC-defined HAIs, IPC-defined outbreak events, and IPC with sequencing-defined outbreak events will be expressed as rate per week per 100 inpatients.

The outcomes of genotypic identification of transmission linkage not suspected at initial IPC investigation and impact of sequencing on IPC actions are only defined for the intervention periods. For such outcomes, the focus of analysis is to calculate summary statistics overall and for each site, which can be informally compared with the degree to which it is thought each site was able to fully implement the intervention. Variation over time within each site will also be explored, and the proportions will be compared between the rapid sequencing and delayed sequencing intervention periods.

The main analyses for the primary and secondary outcomes will be carried out on an intention-to-treat (ITT) basis according to the defined study phases. However, sensitivity analyses will be conducted excluding study sites and/or periods with suboptimal implementation of the study intervention, both in terms of overall population sequencing coverage for HOCIs and the turnaround time for sequence reports being returned to IPC teams.

For outcomes defined in both the baseline and intervention periods, such as incidence of IPC-defined HAIs and the number IPC-defined hospital outbreaks this can be informally compared between the baseline, intervention and (where implementation is justified) final control periods within sites. A formal analysis will be conducted based on negative binomial regression to detect the change in the incidence rate of each event type between baseline, intervention and control phases within site, including the current proportion of inpatients who are SARS-CoV-2 positive as a fixed effect and exposure 'determined' by the number of SARS-CoV-2 negative inpatients in that week. These analyses will also include adjustment for the proportion of HCWs at each site who have received at least one vaccine dose, and a smoothed adjustment for calendar time. This will lead to an adjusted incidence rate ratio for the intervention effect, presented with a 95% confidence interval.

Missing data will be identified, and efforts made to obtain the data. In the event that some sites are unable to implement the intervention fully during the intervention period then analysis will be repeated excluding such sites to provide a 'per protocol' analysis.

For peer review only - http://bmjopen.bmj.com/site/about/guidelines.xhtml

A detailed statistical analysis plan (SAP) will be produced prior to commencement of analysis and agreed by the joint Trial Steering Committee and Data Monitoring Committee (TSC-DMC). All statistical tests will use a 2-sided p-value of 0.05, unless otherwise specified. All statistical analysis will be performed using Stata (StataCorp, College Station, TX, USA).

HEALTH ECONOMIC ANALYSIS

We will examine whether rapid SARS-CoV-2 genomic sequencing might lead to measurable economic advantages. A cost-benefit analysis will be conducted looking at the incremental cost or savings for the two sequencing approaches against baseline in the group of sites influenced by the time to sequence data result.

The cost of SARS-CoV-2 genome sequencing, generating the report and additional resources involved in teams training and review of the report will be obtained from the participating laboratories and study sites.

HOCI resource use will be obtained from hospital records and CRFs supplemented with information obtained from members of the IPC teams at each site to inform IPC action related cost. Costs will be evaluated from the NHS setting perspective over the study period. Economic benefits include the attributable cost savings from reducing the delay to initiate IPC measures to avert infection transmission, an estimate of the hospital cost savings due to excess bed days, and days off work by HCWs.

Mean costs and standard deviations for all phases of the study will be calculated. We will estimate the incremental mean difference in total costs between intervention phases and baseline of the study and 95% confidence intervals.

Deterministic sensitivity analysis will be performed to assess the impact of varying resource use and other relevant parameters to identify variables with the highest impact on costs.

Adjustments will be made for variation in HOCI levels due to impact of B.1.1.7 variant of SARS-CoV-2 in the UK, as well as the national COVID-19 vaccine rollout.

A Health Economic Analysis Plan will be prepared for the study prior to commencing data analysis and will be approved by the TSC-DMC.

For peer review only

For peer review only - http://bmjopen.bmj.com/site/about/guidelines.xhtml

PROCESS EVALUATION

Process evaluations are now considered integral to understanding the factors which shape outcomes achieved within a study, enrich interpretation of findings and facilitate better understandings of how the intervention may be used in other settings to create sustainable health change.

The process evaluation embedded within the HOCI study aims to understand how the rapid genome sequencing intervention works in practice across different sites.

The team will first develop initial programme theory for the SRT in advance of implementation. Programme theory describes the salient parts of the context in which the intervention will be implemented, the specific nature of the problem being addressed, the content or components of the intervention, the mechanisms through which the intervention works and how the intervention led to expected and unanticipated outcomes. Including the development of the programme theory as part of the study ensures that the team have de-risked the intervention as far as possible by anticipating and mitigating potential problems or limiting factors.[8]

Data will then be gathered using a topic guide based on the programme theory. A purposive sample of HCWs involved in the chain of activity associated with implementing the SRT across five study sites will be interviewed. Interviews will take place during or soon after the rapid phase and focus on how the SRT and HOCI study more broadly have been implemented. A structured thematic analysis of the data will be conducted using the core elements of the initial programme theory, which will then be refined and used to share learning on HOCI and facilitate transferable knowledge and sustainable future healthcare.

STUDY TIMELINES

Sites will be opened using a staggered approach from October 2020 to January 2021 in order to provide the greatest likelihood of each phase of site activity covering peaks, troughs and moderate incidences of community prevalence and therefore likely hospital admissions of COVID-19 patients.

Patient recruitment at sites will run for six months from late October 2020 to end April 2021.

See Table 1 for Study Schedule.

For peer review only

DISCUSSION

By defining and reporting SARS-CoV-2 genotype frequencies within its sites and comparing to those in the wider community, the study has the potential to overcome some of the inherent barriers to identifying the likely transmission chains. The data generated will provide an accurate as possible a picture, given the constraints of viral genetic diversity, of the number and location of SARS-CoV-2 infections acquired by nosocomial transmission, and to an extent inform how these transmissions are occurring.

While COG-UK will provide data on the utility of viral genomics for national public health planning, COG-UK HOCI will quantify the utility of the same data for local management of nosocomial infection, including whether observed benefits are time dependent and deliver the best estimates of how viral sequence data can be used to identify HAIs among HOCIs.

Study outputs will further inform decisions about the likely future use of viral genome sequencing for the management of epidemics and pandemics and how it might best be organised - centralised or diversified - to deliver maximal impact.

STUDY MONITORING

An independent joint Trial Steering Committee and Data Monitoring Committee (TSC-DMC) will be formally responsible for the oversight of the study and ensuring it is conducted in compliance with ICH Good Clinical Practice and other relevant regulations. The TSC-DMC will also advise on the need for the fourth phase of the study (a second baseline period).

The Trial Management Group (TMG) will be responsible for the execution of the study. Site monitoring will be undertaken by the Trial Manager, based at the UCL Comprehensive Clinical Trials Unit.

Site teams will only report adverse events which meet both the 'seriousness' threshold and are also considered 'related' to the study intervention. This was considered risk appropriate for the study as no patient-specific procedures are undertaken, and has been approved by the Ethics Committee.

PATIENT AND PUBLIC INVOLVEMENT

The COG-UK HOCI study was designed between April and May 2020 and was initially intended to run during the first wave of the COVID-19 epidemic in the UK, and therefore for timing and

For peer review only - http://bmjopen.bmj.com/site/about/guidelines.xhtml

safety reasons, patients with COVID-19 were not directly included to participate in the study's design development.

ETHICS AND DISSEMINATION

The National Research Ethics Service Committee (Cambridge South) reviewed the study protocol (approved 23rd April 2020, REC ref 20/EE/0118). Any subsequent amendments to these documents will be submitted for further approval. The findings of this study will be disseminated through peer-reviewed publications and conference presentations.

POST-STUDY ACCESS TO DATA

The terms of the funding requires the COG-UK HOCI study dataset to be shared on CSDR (clinicalstudydatarequest.com) or an equivalent data sharing platform so that the data may be reused by other researchers. This will be done within 6 months of public reporting of results.

For peer review only

Table 1 - Study Schedule

Timepoint (site dependent)	6 months	4 weeks	8 weeks	4 weeks	4 weeks	6 Months
**Study stage (site dependent)**	**Set up**	**First Baseline / Control (daily)**	**Intervention sequencing result <48 hours (daily)***	**Intervention sequencing result >4 days (daily)***	**Second Baseline / Control (where justified, daily)**	**Data cleaning, analysis and reporting**
Site identification	X					
Site team discussion on sampling ability, staffing availability, and logistics	X					
Initiation of contracts, R&D approvals	X					
NHS samples begin to be processed locally under COG-UK approvals (not study-related)	X					
NHS samples to be processed under COG-UK approvals, either locally or at Sanger		X	X	X	X	
Intervention reports generated			X	X		
Intervention reports returned to site ICTs (<48h)			X			
Intervention reports returned to site ICTs (>4 days)				X		
ICTs evaluate reports, seeking Expert Sequence Interpretation Team views if needed			X	X		
Case reports for HOCIs		X	X	X	X	
Process evaluation - qualitative interviews /analysis			X	X	X	X
Process evaluation - programme theory development and refinement	X		X	X	X	X
Interim analysis and views from TSC-DMC whether second baseline/control state acceptable					X [A]	
Data cleaning		X	X	X	X	X
Final data lock and analysis						X
Reporting/publication						X

* The order of 'rapid' phase (<48 hour turnaround time) and 'slow' phase may be swapped prior to commencement of either upon agreement with the Sponsor. The option is offered to facilitate logistics/set-up at sites.

[A] TSC-DMC review should take place to determine whether it would be considered ethical to request sites have a second period of baseline/control (where sequencing data is not provided to IPC teams). This would only be on the basis that it is unclear from the initial baseline and intervention comparison whether there is a significant benefit; in cases where it is clear there is either benefit to the intervention or no benefit, then the second baseline would not take place.

For peer review only - http://bmjopen.bmj.com/site/about/guidelines.xhtml

Figure 1 – Example of HOCI Sequence Reporting Tool (SRT) Report Output

[Figure 1 – SRT Report.tif image about here]

For peer review only

ACKNOWLEDGEMENTS

Our thanks to the Principal Investigators: Dr Anu Chawla (Liverpool University Hospitals NHS Foundation Trust); Dr Maria-Teresa Cutino-Moguel (Barts Health NHS Trust); Prof Alison Holmes (Imperial College Healthcare NHS Trust); Dr Nick Machin (Manchester University NHS Foundation Trust); Dr Nikunj Mahida (Nottingham University Hospitals NHS Trust); Dr Taibtha Mahungu (Royal Free NHS Foundation Trust); Dr Gaia Nebbia (Guy's and St Thomas' NHS Foundation Trust); Dr Cassie Pope (St George's University Hospitals NHS Foundation Trust); Dr Kordo Saeed (University Hospital Southampton NHS Foundation Trust); Dr Tranprit Saluja (Sandwell and West Birmingham NHS Trust); Dr Gee Yen Shin (University College London Hospitals NHS Foundation Trust); Dr Thushan de Silva (Sheffield Teaching Hospitals NHS Foundation Trust); Dr Yusri Taha (Newcastle Hospitals NHS Foundation Trust); Prof Emma Thomson (NHS Glasgow and Greater Clyde) and teams for their time and support.

We also acknowledge the support of the independent members of the Joint Trial Steering Committee and Data Monitoring Committee (TSC-DMC):

Prof Marion Koopmans (Erasmus MC)

Prof Walter Zingg (University of Geneva)

Prof Colm Bergin (Trinity College Dublin)

Prof Karla Hemming (University of Birmingham)

Prof Katherine Fielding (LSHTM)

As well as TSC-DMC non-independent members:

Prof Nick Lemoine (NIHR CRN)

Prof Sharon Peacock (COG-UK)

We would also thank members of COG-UK who have directly supported the study:

Dr Ewan Harrison (Cambridge University)

Dr Katerina Galai (PHE)

Dr Francesc Coll (LSHTM)

Dr Michael Chapman (HDR-UK)

Prof Thomas Connor and team (Cardiff University)

Prof Nick Loman and team (University of Birmingham)

For peer review only

We also thank the COG-UK Consortium, and the UK National Institute for Health Research Clinical Research Network (NIHR CRN).

COLLABORATORS

MRC–University of Glasgow Centre for Virus Research (CVR), Glasgow, UK

Dr Joshua Singer, Dr Joseph Hughes and Prof Emma Thomson in supporting design and implementation of the HOCI sequence reporting tool.

University of Sheffield, Sheffield, UK

Dr Matthew Parker supporting design and testing of the HOCI sequence reporting tool.

UCL, London, UK

Dr Asif Tamuri, Dr Sunando Roy and Dr Stefan Piatek supporting design, implementation and testing of the HOCI sequence reporting tool.

CONTRIBUTORS

JBl developed the study protocol and contributed to the writing of the manuscript. OS and AC were involved in development of the statistical analysis facets of the trial and contributed to the writing of the manuscript. MP was involved in development of the health economic analysis facets of the study and contributed to the writing of the manuscript. PF and FM were involved in development of the process evaluation of the study and contributed to the writing of the manuscript. LH is the trial manager for the study. JP, PD, CP, TS, GN and LS are responsible for the operationalisation of study procedures. RM will contribute to data collection and management. JBr is the chief investigator for the study. All authors critically reviewed and approved the final version.

FUNDING

The COG-UK HOCI study is funded by the COG-UK Consortium, which is supported by funding from the UKRI MRC Grant number MC PC 4050788737.

SPONSOR

The COG-UK HOCI study is sponsored by University College London, represented by the Comprehensive Clinical Trials Unit (UCL CCTU).

COMPETING INTERESTS

None declared.

For peer review only

REFERENCES

1. Roy S, Hartley J, Dunn H et al. Whole-genome Sequencing Provides Data for Stratifying Infection Prevention and Control Management of Nosocomial Influenza A. *Clin Infect Dis* 2019; 69, 1649-1656, doi:10.1093/cid/ciz020.

2. Houldcroft C, Roy S, Morfopoulou S et al. Use of Whole-Genome Sequencing of Adenovirus in Immunocompromised Pediatric Patients to Identify Nosocomial Transmission and Mixed-Genotype Infection. *J Infect Dis* 2018; 218, 1261-1271, doi:10.1093/infdis/jiy323

3. Brown J, Roy S, Shah D et al. Norovirus Transmission Dynamics in a Pediatric Hospital Using Full Genome Sequences. *Clin Infect Dis* 2019; 68, 222-228, doi:10.1093/cid/ciy438

4. Bedford, T. Phylodynamic estimation of incidence and prevalence of novel 362 coronavirus (nCoV) infections through time. *Virological*, accessed on 27/02/2020. http://virological.org/t/phylodynamic-estimation-of-incidence-and-prevalence-of-novel364 coronavirus-ncov-infections-through-time/391

5. COVID-19 Genomics UK (COG-UK) consortium. An integrated national scale SARS-CoV-2 genomic surveillance network. *Lancet Microbe* 1(3), 2020; 99-100, https://doi.org/10.1016/S2666-5247(20)30054-9

6. Stirrup O, Hughes J, Parker M et al. Rapid feedback on hospital onset SARS-CoV-2 infections combining epidemiological and sequencing data. accessed on 16/03/2021 medRxiv 2020.11.12.20230326; doi: https://doi.org/10.1101/2020.11.12.20230326

7. Flowers P, Mapp F, Blackstone J et al. Developing Initial Programme Theory: COVID-19 Genomics UK Consortium Hospital-onset COVID-19 Study (COG-UK HOCI). SocArXiv. Accessed on 10/02/2021. doi:10.31235/osf.io/ysm35.

8. Public Health England. COVID-19: epidemiological definitions of outbreaks and clusters. Gov.uk, accessed on 03/04/2021. https://www.gov.uk/government/publications/covid-19-epidemiological-definitions-of-outbreaks-and-clusters/covid-19-epidemiological-definitions-of-outbreaks-and-clusters-in-particular-settings

For peer review only

COG-UK HOCI Summary Report

Focus sample [redacted]

Report date	21-Sep-2020	Unit	Unit_[redacted]
Sample ID	-	Previous unit(s)	
Sample date		Hospital	Hospital_[redacted]
COG-UK HOCI ID	-	Reporting hub	-
COG-UK ID		Reported by	-
Admission date			

Report

Lineage: B.1.p73

Focus patient's sample sequence is closely matched to samples below, possibly linked by transmission.

⚠ Infection within the unit is very highly probable* ⚠

Number	Sample ID	COG-UK ID	Previous unit(s)	Sample date	Admission date	Type
1	-					Patient
2	-					Patient
3	-					Patient
4	-					Patient
5	-					Patient

Infection within the hospital has low probability*

Number	Sample ID	COG-UK ID	Unit	Previous unit(s)	Sample date	Admission date	Type
6	-						Patient
7	-						Patient

Please check IPC data, and PATIENT and HCW movement, particularly for the 10-14 days preceding the date of the focus patient's sample.

- Infection from a visitor has low probability*
- Community-acquired infection has low probability*

** likelihood of transmission risk: 0-30% low ; 30-50% moderately low; 50-70% probable; 70-85% high; 85-100% very high*

Timeline

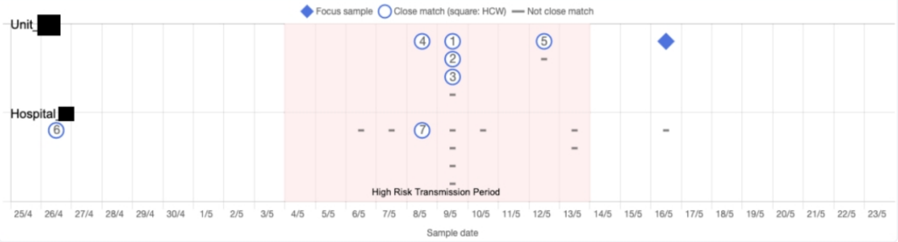

Generated on: 21-Sep-2020 CoV-GLUE version: 0.1.13 HOCI version: 0.1.9

Figure 1. Sequence Reporting Tool (SRT) example

SPIRIT
STANDARD PROTOCOL ITEMS: RECOMMENDATIONS FOR INTERVENTIONAL TRIALS

SPIRIT 2013 Checklist: Recommended items to address in a clinical trial protocol and related documents*

Section/item	Item No	Description	Addressed on page number
**Administrative information**			
Title	1	Descriptive title identifying the study design, population, interventions, and, if applicable, trial acronym	1
Trial registration	2a	Trial identifier and registry name. If not yet registered, name of intended registry	3
	2b	All items from the World Health Organization Trial Registration Data Set	3
Protocol version	3	Date and version identifier	2
Funding	4	Sources and types of financial, material, and other support	20
Roles and responsibilities	5a	Names, affiliations, and roles of protocol contributors	20
	5b	Name and contact information for the trial sponsor	20
	5c	Role of study sponsor and funders, if any, in study design; collection, management, analysis, and interpretation of data; writing of the report; and the decision to submit the report for publication, including whether they will have ultimate authority over any of these activities	20
	5d	Composition, roles, and responsibilities of the coordinating centre, steering committee, endpoint adjudication committee, data management team, and other individuals or groups overseeing the trial, if applicable (see Item 21a for data monitoring committee)	19
**Introduction**			
Background and rationale	6a	Description of research question and justification for undertaking the trial, including summary of relevant studies (published and unpublished) examining benefits and harms for each intervention	4
	6b	Explanation for choice of comparators	5
Objectives	7	Specific objectives or hypotheses	5

For peer review only

For peer review only - http://bmjopen.bmj.com/site/about/guidelines.xhtml

Trial design	8	Description of trial design including type of trial (eg, parallel group, crossover, factorial, single group), allocation ratio, and framework (eg, superiority, equivalence, noninferiority, exploratory)	5

Methods: Participants, interventions, and outcomes

Study setting	9	Description of study settings (eg, community clinic, academic hospital) and list of countries where data will be collected. Reference to where list of study sites can be obtained	8
Eligibility criteria	10	Inclusion and exclusion criteria for participants. If applicable, eligibility criteria for study centres and individuals who will perform the interventions (eg, surgeons, psychotherapists)	8
Interventions	11a	Interventions for each group with sufficient detail to allow replication, including how and when they will be administered	6
	11b	Criteria for discontinuing or modifying allocated interventions for a given trial participant (eg, drug dose change in response to harms, participant request, or improving/worsening disease)	N/A
	11c	Strategies to improve adherence to intervention protocols, and any procedures for monitoring adherence (eg, drug tablet return, laboratory tests)	N/A
	11d	Relevant concomitant care and interventions that are permitted or prohibited during the trial	N/A
Outcomes	12	Primary, secondary, and other outcomes, including the specific measurement variable (eg, systolic blood pressure), analysis metric (eg, change from baseline, final value, time to event), method of aggregation (eg, median, proportion), and time point for each outcome. Explanation of the clinical relevance of chosen efficacy and harm outcomes is strongly recommended	9-10
Participant timeline	13	Time schedule of enrolment, interventions (including any run-ins and washouts), assessments, and visits for participants. A schematic diagram is highly recommended (see Figure)	17
Sample size	14	Estimated number of participants needed to achieve study objectives and how it was determined, including clinical and statistical assumptions supporting any sample size calculations	10-11
Recruitment	15	Strategies for achieving adequate participant enrolment to reach target sample size	8

Methods: Assignment of interventions (for controlled trials)

Allocation:			N/A

For peer review only - http://bmjopen.bmj.com/site/about/guidelines.xhtml

Sequence generation	16a	Method of generating the allocation sequence (eg, computer-generated random numbers), and list of any factors for stratification. To reduce predictability of a random sequence, details of any planned restriction (eg, blocking) should be provided in a separate document that is unavailable to those who enrol participants or assign interventions	
Allocation concealmen t mechanism	16b	Mechanism of implementing the allocation sequence (eg, central telephone; sequentially numbered, opaque, sealed envelopes), describing any steps to conceal the sequence until interventions are assigned	N/A
Implementat ion	16c	Who will generate the allocation sequence, who will enrol participants, and who will assign participants to interventions	N/A
Blinding (masking)	17a	Who will be blinded after assignment to interventions (eg, trial participants, care providers, outcome assessors, data analysts), and how	N/A
	17b	If blinded, circumstances under which unblinding is permissible, and procedure for revealing a participant's allocated intervention during the trial	N/A

Methods: Data collection, management, and analysis

Data collection methods	18a	Plans for assessment and collection of outcome, baseline, and other trial data, including any related processes to promote data quality (eg, duplicate measurements, training of assessors) and a description of study instruments (eg, questionnaires, laboratory tests) along with their reliability and validity, if known. Reference to where data collection forms can be found, if not in the protocol	11
	18b	Plans to promote participant retention and complete follow-up, including list of any outcome data to be collected for participants who discontinue or deviate from intervention protocols	N/A
Data management	19	Plans for data entry, coding, security, and storage, including any related processes to promote data quality (eg, double data entry; range checks for data values). Reference to where details of data management procedures can be found, if not in the protocol	11
Statistical methods	20a	Statistical methods for analysing primary and secondary outcomes. Reference to where other details of the statistical analysis plan can be found, if not in the protocol	12
	20b	Methods for any additional analyses (eg, subgroup and adjusted analyses)	12-13
	20c	Definition of analysis population relating to protocol non-adherence (eg, as randomised analysis), and any statistical methods to handle missing data (eg, multiple imputation)	12

Methods: Monitoring

For peer review only - http://bmjopen.bmj.com/site/about/guidelines.xhtml

Data monitoring	21a	Composition of data monitoring committee (DMC); summary of its role and reporting structure; statement of whether it is independent from the sponsor and competing interests; and reference to where further details about its charter can be found, if not in the protocol. Alternatively, an explanation of why a DMC is not needed	19
	21b	Description of any interim analyses and stopping guidelines, including who will have access to these interim results and make the final decision to terminate the trial	N/A
Harms	22	Plans for collecting, assessing, reporting, and managing solicited and spontaneously reported adverse events and other unintended effects of trial interventions or trial conduct	15
Auditing	23	Frequency and procedures for auditing trial conduct, if any, and whether the process will be independent from investigators and the sponsor	N/A
**Ethics and dissemination**			
Research ethics approval	24	Plans for seeking research ethics committee/institutional review board (REC/IRB) approval	16
Protocol amendments	25	Plans for communicating important protocol modifications (eg, changes to eligibility criteria, outcomes, analyses) to relevant parties (eg, investigators, REC/IRBs, trial participants, trial registries, journals, regulators)	N/A
Consent or assent	26a	Who will obtain informed consent or assent from potential trial participants or authorised surrogates, and how (see Item 32)	8-9
	26b	Additional consent provisions for collection and use of participant data and biological specimens in ancillary studies, if applicable	N/A
Confidentiality	27	How personal information about potential and enrolled participants will be collected, shared, and maintained in order to protect confidentiality before, during, and after the trial	9
Declaration of interests	28	Financial and other competing interests for principal investigators for the overall trial and each study site	20
Access to data	29	Statement of who will have access to the final trial dataset, and disclosure of contractual agreements that limit such access for investigators	16
Ancillary and post-trial care	30	Provisions, if any, for ancillary and post-trial care, and for compensation to those who suffer harm from trial participation	N/A
Dissemination policy	31a	Plans for investigators and sponsor to communicate trial results to participants, healthcare professionals, the public, and other relevant groups (eg, via publication, reporting in results databases, or other data sharing arrangements), including any publication restrictions	16
	31b	Authorship eligibility guidelines and any intended use of professional writers	20

For peer review only

For peer review only - http://bmjopen.bmj.com/site/about/guidelines.xhtml

	31c	Plans, if any, for granting public access to the full protocol, participant-level dataset, and statistical code	N/A
**Appendices**			
Informed consent materials	32	Model consent form and other related documentation given to participants and authorised surrogates	N/A
Biological specimens	33	Plans for collection, laboratory evaluation, and storage of biological specimens for genetic or molecular analysis in the current trial and for future use in ancillary studies, if applicable	N/A

*It is strongly recommended that this checklist be read in conjunction with the SPIRIT 2013 Explanation & Elaboration for important clarification on the items. Amendments to the protocol should be tracked and dated. The SPIRIT checklist is copyrighted by the SPIRIT Group under the Creative Commons "Attribution-NonCommercial-NoDerivs 3.0 Unported" license.

For peer review only

For peer review only - http://bmjopen.bmj.com/site/about/guidelines.xhtml

BMJ Open

Protocol for the COG-UK hospital onset COVID-19 infection (HOCI) multicentre interventional clinical study: evaluating the efficacy of rapid genome sequencing of SARS-CoV-2 in limiting the spread of COVID-19 in United Kingdom NHS hospitals

Journal:	*BMJ Open*
Manuscript ID	bmjopen-2021-052514.R1
Article Type:	Protocol
Date Submitted by the Author:	17-Feb-2022
Complete List of Authors:	Blackstone, James; University College London, Comprehensive Clinical Trials Unit<br>Stirrup , Oliver ; University College London, Institute for Global Health<br>Mapp, Fiona; UCL, Institute for Global Health<br>Panca, Monica; University College London, Comprehensive Clinical Trials Unit<br>Copas, Andrew; University College London, Institute for Global Health<br>Flowers, Paul; University of Strathclyde<br>Hockey, Leanne; UCL, Comprehensive Clinical Trials Unit<br>Price , James ; Imperial College London, Department of Infectious Disease<br>Partridge, David; Sheffield Teaching Hospitals NHS Foundation Trust, Department of Virology<br>Peters, Christine; NHS Greater Glasgow and Clyde<br>de Silva, Thushan; The University of Sheffield, Department of Infection, Immunity and Cardiovascular Disease<br>Nebbia, Gaia; Guy's and St Thomas' NHS Foundation Trust, Department of Infectious Diseases<br>Snell, Luke; Guy's and St Thomas' NHS Foundation Trust, Department of Infectious Diseases<br>McComish, Rachel; University College London, Comprehensive Clinical Trials Unit<br>Breuer, Judith; UCL
**Primary Subject Heading**:	Infectious diseases
Secondary Subject Heading:	Genetics and genomics, Epidemiology
Keywords:	COVID-19, Infection control < INFECTIOUS DISEASES, MOLECULAR BIOLOGY, Epidemiology < INFECTIOUS DISEASES

For peer review only - http://bmjopen.bmj.com/site/about/guidelines.xhtml

For peer review only - http://bmjopen.bmj.com/site/about/guidelines.xhtml

Protocol for the COG-UK hospital onset COVID-19 infection (HOCI) multicentre interventional clinical study: evaluating the efficacy of rapid genome sequencing of SARS-CoV-2 in limiting the spread of COVID-19 in United Kingdom NHS hospitals

Authors: James Blackstone[1]; Oliver Stirrup[2]; Fiona Mapp[2]; Monica Panca[1]; Andrew Copas[2]; Paul Flowers[3]; Leanne Hockey[1]; James Price[4]; David Partridge[5]; Christine Peters[6]; Thushan de Silva[7]; Gaia Nebbia[8]; Luke B. Snell[8]; Rachel McComish[1]; the COVID-19 Genomics UK (COG-UK) Consortium; Judith Breuer[9].

1 Comprehensive Clinical Trials Unit, Institute of Clinical Trials and Methodology, UCL, London, UK
2 Institute for Global Health, UCL, London, UK
3 School of Psychological Sciences and Health, University of Strathclyde, Glasgow, UK
4 Faculty of Medicine, Department of Infectious Disease, Imperial College London, UK
5 Sheffield Teaching Hospitals NHS Foundation Trust, Sheffield, UK
6 Queen Elizabeth University Hospital, Greater Glasgow and Clyde Heath Board, Glasgow, UK
7 Department of Infection, Immunity and Cardiovascular Disease, University of Sheffield, Sheffield, UK
8 Department of Infectious Diseases, Guy's and St Thomas' NHS Foundation Trust, London, UK
9 Infection, Immunity and Inflammation, Great Ormond Street Institute of Child Health, UCL, London, UK

Word count: 4,267

Corresponding Author:
Prof. Judith Breuer, j.breuer@ucl.ac.uk
Institute of Child Health, University College London, 30 Guilford Street, London, WC1N 1EH, UK

For peer review only

ABSTRACT

Objectives

Nosocomial transmission of severe acute respiratory syndrome coronavirus 2 (SARS-CoV-2) has been a significant cause of mortality in National Health Service (NHS) hospitals during the coronavirus disease 2019 (COVID-19) pandemic. The COG-UK HOCI (COG-UK Consortium Hospital-Onset COVID-19 Infections) study aims to evaluate whether the use of rapid whole genome sequencing of SARS-CoV-2, supported by a novel probabilistic reporting methodology, can inform infection prevention and control (IPC) practice within NHS hospital settings.

Design

Multicentre, prospective, interventional, superiority study.

Setting

14 participating NHS hospitals over winter-spring 2020/2021 in the United Kingdom.

Participants

Eligible patients must be admitted to hospital with first confirmed SARS-CoV-2 PCR positive test result >48h from time of admission, where COVID-19 diagnosis not suspected upon admission. The projected sample size is 2,380 patients.

Intervention

The intervention is the return of a sequence report, within 48 hours in one phase (rapid local lab processing) and within 5-10 days in a second phase (mimicking central lab), comparing the viral genome from an eligible study participant with others within and outside the hospital site.

Primary and secondary outcome measures

The primary outcomes are incidence of Public Health England (PHE)/IPC-defined SARS-CoV-2 hospital-acquired infection during the baseline and two interventional phases, and proportion of hospital-onset cases with genomic evidence of transmission linkage following implementation of the intervention where such linkage was not suspected by initial IPC investigation. Secondary outcomes include incidence of hospital outbreaks, with and without sequencing data; actual and desirable changes to IPC actions; periods of healthcare worker

For peer review only

(HCW) absence. Health economic analysis will be conducted to determine cost-benefit of the intervention. A process evaluation using qualitative interviews with HCWs will be conducted alongside the study.

Study Registration

ISRCTN50212645. This manuscript is based on protocol version 6.0. 02-Sep-21

Strengths and limitations of this study

- Harnesses infrastructure of UK's existing national COVID-19 genome sequencing platform.
- First prospective interventional study to assess effectiveness of genomic sequencing for IPC in an unbiased patient selection in secondary care.
- Awarded UK NIHR Urgent Public Health status, ensuring prioritisation of recruitment.
- A limitation is that the study does not have a randomised controlled design.

For peer review only

For peer review only - http://bmjopen.bmj.com/site/about/guidelines.xhtml

INTRODUCTION

Background

Hospitals are recognised to be a major risk for the spread of infections despite the universal introduction of infection control measures. For severe acute respiratory syndrome coronavirus 2 (SARS-CoV-2), nosocomial spread of infection presents an additional and significant health risk to patients and healthcare workers (HCW).[1] During epidemics, infection prevention and control (IPC) practice is further complicated by the difficulties of distinguishing community and hospital-acquired infections. This can lead to erroneous identification of nosocomial transmission leading to unnecessary IPC efforts. True nosocomial transmission events may be missed with appropriate interventions not performed, thereby putting patients and HCW at increased risk. The epidemiological determination of infection timing for SARS-CoV-2 is made especially challenging by its prolonged incubation period in distinguishing community from nosocomial transmission.

There is now good evidence that genome sequencing of epidemic viruses, together with standard IPC, better defines nosocomial transmissions and, depending on the virus, better identifies routes of transmission, than IPC alone.[2-4] The development of rapid sequencing methods enable sequencing of potentially linked or unlinked SARS-CoV-2 genomes within 48 hours. This timescale is short enough to inform clinically relevant IPC decisions in near-real-time. Although some studies have described the prospective use of viral sequencing to inform infection control for SARS-CoV-2, none have prospectively evaluated the impact of sequencing on the incidence of nosocomial infection or on infection control actions across all cases with hospital onset.[5-7]

While SARS-CoV-2 has a low mutation rate (estimated at around 2.5 changes per genome per month), sufficient viral diversity exists to identify cases where patient and HCW infections that are clustered in time and space are in fact due to different SARS-CoV-2 genotypes.[8] Such information could rapidly exclude nosocomial transmission as the cause of the cluster and redirect IPC intervention to where needed most.

Confirmation of SARS-CoV-2 transmission to patients and healthcare workers may be more challenging with a single observed mutation between two genomes, feasibly representing anything between one and ten transmissions. Identical genomes will not necessarily evidence a close link between two cases. Nonetheless, by comparing genotypes detected within the hospital setting and the surrounding community, it may be possible to reveal unsuspected

For peer review only - http://bmjopen.bmj.com/site/about/guidelines.xhtml

nosocomial transmission where comparatively uncommon genotypes are apparently linked or cluster in time and space.

The COG-UK initiative aims to sequence as many SARS-CoV-2 viruses as possible across the UK for public health planning. It also provides an important and unique opportunity to test whether viral sequence data produced in near-real-time could also reduce uncertainties around nosocomial transmission events, better direct IPC effort, improve hospital functioning and reduce the role of hospitals as a source of infection to the community. [9]

COG-UK HOCI* will harness the COG–UK sequencing platform, with its mixed model of smaller sequencing hubs located close to hospitals and a large centralised hub sequencing most viruses. It will identify not only whether rapid viral sequencing is useful for patient management, but how time-critical this might be; turnaround times for sequence data from a central hub are likely to be longer (5-10 days) than those from local sequencing hubs (<48 hours).

*Note that while 'hospital-onset COVID-19 infections' (HOCI) was the preferred term at the study's inception, evolution of the terminology now favours references be made to 'hospital-onset SARS-CoV-2 infections.'

Objective

The study will evaluate the contribution of whole-genome sequencing combined with a novel viral sequence report design to IPC investigation and response to cases of hospital onset coronavirus disease 2019 (COVID-19) infection, and whether this can reduce the overall incidence of hospital-acquired infections. [10]

METHODS AND ANALYSIS

Study design

COG-UK HOCI is a prospective, interventional, superiority clinical study, comprising three distinct phases with a possible fourth, dependant on interim data analysis.

In the first phase, all sites will collect baseline (non-interventional) eligible patient data for a period of four weeks to characterise each site's usual practice in infection control in response to hospital-onset COVID-19 cases. This phase may include standard of care use of genome sequencing (e.g. limited outbreak response analysis).

For peer review only - http://bmjopen.bmj.com/site/about/guidelines.xhtml

In the second and third phases, the study intervention will be applied on top of standard of care infection control practices.

The second phase requires ‘rapid’ turnaround of genome sequencing and sequence linkage report generation (i.e. within 48 hours of first diagnostic SARS-CoV-2 polymerase chain reaction positive result). This phase will be applied to all hospital onset COVID-19 cases meeting the eligibility criteria over an 8 week period.

The third phase is similar to the second, except that a 5-10 day turnaround time of genome sequencing and sequence report generation should be applied to mimic the use of a central sequencing laboratory. This phase will apply to all sites and last for 4 weeks.

The second and third phases may be applied in the reverse order at some sites, both for logistical reasons (i.e. fine-tuning of rapid turnaround of whole-genome sequences) and also to ensure differences between sites in the calendar dates of each phase.

Upon review of interim analysis data, the study’s joint oversight committee may recommend a fourth phase for all sites comprising a second baseline period; this would be applied where the initial baseline data collection period occurred at a time of very high or low COVID-19 prevalence at the sites, whereby collecting data on standard practice could be unviable.

This will be a sequential study, with each NHS Trust acting as its own control.

The total study duration per site will accordingly be 16-20 weeks, though it is likely that pauses in data collection will occur over the winter holiday break period due to most sequencing labs closing or moving to skeletal operations during this time.

For peer review only

INTERVENTION

Overview

The study intervention is a SARS-CoV-2 genomic sequencing data report (see Figure 1) delivered to the NHS site’s Infection Prevention and Control (IPC) teams, either within 24-48 hours of the sample from the patient being confirmed as positive for SARS-CoV-2 (rapid genomic sequencing locally), or within 5-10 days (local genomic sequencing to mimic use of a centralised lab). [11]

Microbiology and IPC teams will be trained to interpret the results. An expert sequence interpretation team (a sub-set of the Study Team) will be available seven days a week by

phone and online to discuss results where required with IPC teams, and to provide guidance on best practice.

Genomic sequence reporting tool

The genomic sequence report tool combines epidemiological and consensus sequence data in order to provide a rapid assessment of the probability of hospital acquired infection (HAI) among new HOCI study cases and to identify infections that could plausibly constitute a hospital outbreak event. [12]

The internal calculations use a combination of admission-to-symptom onset intervals and differences between the observed proportion of close sequence matches (defined as a maximum pairwise difference of 2 single nucleotide polymorphisms; SNPs) for viral samples obtained from various locations (i.e. same ward, same hospital, within the community) to estimate the probability that the patient's SARS-CoV-2 infection was acquired in hospital.

The report generation algorithm is designed to run quickly and reliably, without the need for local model checking, thereby reducing the need for expert bioinformatics input during operation.

Sequence reports

The summary report for each focus sequence submitted, corresponding to a single HOCI case, will comprise:

1. The lineage assignment for the focus sequence.
2. A list of the details of any close sequence matches from samples on the same ward as the focus sequence in the previous three weeks, with estimated probability of infection having occurred from a source on the ward (reported as low, moderately low, probable, high, very high).
3. A list of the details of any close sequence matches from samples obtained within the hospital but not same ward as the focus sequence in the previous three weeks, with estimated probability of infection having occurred from a source in the institution (reported as low, moderately low, probable, high, very high).
4. Estimated probability of infection from a visitor to the ward (reported as low, moderately low, probable, high, very high).
5. Estimated probability of community-acquired infection (reported as low, moderately low, probable, high, very high).

For peer review only - http://bmjopen.bmj.com/site/about/guidelines.xhtml

6. A graphical summary displaying sample dates of close sequence matches at the ward and hospital levels, along with the total number of samples obtained over the previous three weeks.

A detailed report will also be returned to virology labs for each focus sequence, containing additional details of all the recent sequences obtained within the given ward and hospital that have contributed to the output summarised in the summary report, and their similarity with respect to the focus sequence.

Allocation of intervention

All sites will engage in the various study phases sequentially; there will be no allocation of intervention either by site or at patient-level for this study.

POPULATION

Setting

Fourteen NHS Trusts/Heath Boards across England and Scotland will participate. Sites will be set up either as all hospitals within a Trust or a single hospital selected from within the Trust. This decision will be site-led and based on available research team, infection control team and sequencing resource. Sites will be selected to span tertiary referral centres through to district hospitals, primarily in urban or suburban settings. Screening and routine testing of patients and HCWs will follow local health guidance.

Inclusion criteria

Patients will be considered eligible only where they are an inpatient with first confirmed positive test for SARS-CoV-2 >48 hours after admission, where they were not suspected to have COVID-19 at time of admission.

Participants may be of any age to be included in the study.

There are no exclusion criteria.

Recruitment

Viral sequencing will be attempted for every confirmed case of SARS-CoV-2 in hospital patients and HCW, but it is not possible to assess clinical and infection control outcomes for

For peer review only

For peer review only - http://bmjopen.bmj.com/site/about/guidelines.xhtml

every confirmed case. This study will therefore focus on the subset of patients with hospital-onset SARS-CoV-2, since this is where the additional knowledge potentially provided by viral sequencing is likely to have the greatest impact for IPC teams. HCWs will not be enrolled as index cases but will be part of the reference sequence dataset where available. Patient samples will be collected and sequenced per standard NHS Trust practice in support of pre-existing site arrangements to provide SARS-CoV-2 sequence data to COG-UK in support of national genomic surveillance for public health.

Patient consent

Consent for participant (both patient and healthcare worker) involvement will not be sought for COG-UK HOCI study. This approach relies on the Health Service (Control of Patient Information) Regulations 2002 (SI 1438), specifically Regulation 3 (Communicable disease and other risks to public health), and Regulation 7 (the processing of confidential information for medical research). This approach was reviewed and approved by a Research Ethics Committee.

STUDY OUTCOMES

Primary outcomes

1. Incidence rate of PHE/IPC-defined SARS-CoV-2 HAIs (defined as SARS-CoV-2 cases with an interval of ≥8 days from admission to symptom onset, if known, or sample date), measured as incidence rate of recorded cases per week per 100 inpatients, during each phase of the study.
2. Identification of linkage to individuals within an outbreak of SARS-CoV-2 nosocomial transmission using sequencing report data for HOCIs in whom this was not identified by pre-sequencing IPC evaluation, for each enrolled patient during study phases in which the sequence reporting tool is in use.

Secondary outcomes

1. Incidence rate of IPC-defined SARS-CoV-2 hospital outbreaks, defined as cases of hospital transmission linked by location and with intervals between diagnoses no greater than 28 days, measured as incidence rate of outbreak events per week per 100 inpatients during each phase of the study.

For peer review only

For peer review only - http://bmjopen.bmj.com/site/about/guidelines.xhtml

2. Incidence rate of IPC+sequencing-defined SARS-CoV-2 hospital outbreaks involving HOCI cases, defined as for IPC-defined SARS-CoV-2 hospital outbreaks with the additional condition of clustering of viral sequences and measured as outbreak events per week per 100 inpatients during study phases in which the sequence reporting tool is in use. Genetic clusters are defined as having maximum viral sequence pairwise SNP distance of 2 between each individual included and their nearest neighbour within the cluster.
3. Changes to IPC actions implemented following receipt of SARS-CoV-2 sequence report, for each enrolled patient during study phases in which the sequence reporting tool is in use.
4. Changes to IPC actions that would ideally have been implemented but may not have been following receipt of SARS-CoV-2 sequence report, for each enrolled patient during study phases in which the sequence reporting tool is in use.
5. Health economic benefit of both slow and rapid sequencing reports to IPC against baseline.
6. The number of HCW periods of sickness/self-isolation, assessed as a proportion of the number of staff usually on those wards impacted by HOCI cases, for all phases of the study.

In collaboration with IPC teams at NHS sites, the outcome data above will be collected using CRFs specifically designed for this study. These will be prospectively completed during the course of the study and stored in a central study database.

Exploratory outcomes

Additionally, descriptive summaries of sequence report results will be generated, including number of close sequence matches on ward and within hospital; probability of infection source; whether healthcare workers are reported within close sequence matches.

For the process evaluation, the qualitative team will seek to understand how the intervention worked in practice across a representative sample of study sites (n=5). This will include how the context shaped the intervention; how key intervention components and causal mechanisms operated for IPC teams and hospital planners, and how the intervention changed the study outcomes.

For peer review only

For peer review only - http://bmjopen.bmj.com/site/about/guidelines.xhtml

SAMPLE SIZE AND POWER

The projected total sample size is 2,380 patients.

There is uncertainty in the number of HOCIs that will be identified at each site during each of the intervention periods, with the rapid testing phase being eight weeks' duration. Based on clinical experience of first wave and discussion with the principal investigators, we assume there may be an average of 10 HOCIs/week per site during this intervention period, a total of 80 per site. Within a typical site this will allow us to estimate the proportion of HOCIs with genotypic linkage to any other case(s) not detected by IPC processes with minimum precision of +/- 9.4%. Similarly, we can estimate the proportion of HOCIs where an action is taken that would not have occurred without sequencing within +/- 9.4%. We shall also calculate pooled estimates of these proportions across the 14 sites, leading to estimation within +/- 6.5% assuming an intracluster correlation coefficient of 0.05.

Comparing the proportion of HOCIs with genotypic linkage to any other case(s) not detected by IPC processes between rapid testing and slow testing phases across all sites, the study would have at least 80% power to detect a percentage point difference of 11%. This corresponds to a two-sided test with alpha=0.05, considering proportions of 55.5% vs 44.5% which would be associated with minimum power for a difference of this magnitude.

For the outcome of weekly incidence of IPC-defined HOCIs, using an approximate Normal distribution for weekly counts there is 86.7% power to demonstrate a reduction from 12 IPC-defined HOCIs per week in the baseline phase to 10 per week during the rapid testing phase across all sites, under 5% significance level two-tailed testing. However, these calculations correspond to a variance of 12 for weekly counts based on the Poisson distribution, but the presence of over-dispersion of weekly counts would lead to a lower power to detect a difference. Using an overdispersion parameter of 0.82 based on retrospective analysis of data from Sheffield and Glasgow (dataset as described by Stirrup et al.) results in 81% power to detect a reduction in mean weekly incidence from 12.5 to 10. [10]

DATA MANAGEMENT AND PROTECTION

All study documentation at site will be held in restricted access areas and stored securely by study team members. Data will be entered by sites into a secure, validated online database (Elsevier MACRO v4) and accessible only by delegated team members of that site, and by delegated staff from the coordinating centre.

For peer review only - http://bmjopen.bmj.com/site/about/guidelines.xhtml

Case Report Forms (CRFs) for the study will identify patients using a unique five-digit study identifier, year of birth, and initials. Under the Data Protection Act 2018, the latter identifiers will be considered 'personally identifying' and will be treated as such by both the site team and coordinating centre team.

Where written communication (e.g. data queries) on individual patient cases is necessitated between sites and the coordinating centre, only the study identifier should be used in the first instance.

Any transfer of documentation containing personally identifying data between site and coordinating centre will be subject to AES-256 industry-standard encryption.

For peer review only

STATISTICAL ANALYSIS

Summary statistics will be presented for each study phase (baseline, rapid local lab, and central lab interventions) and each site, which will be percentages for binary outcomes such as whether transmission linkage for each HOCI was previously undetected. The frequency of IPC-defined HAIs, IPC-defined outbreak events, and IPC with sequencing-defined outbreak events will be expressed as rate per week per 100 inpatients.

The outcomes of genotypic identification of transmission linkage not suspected at initial IPC investigation and impact of sequencing on IPC actions are only defined for the intervention periods. For such outcomes, the focus of analysis is to calculate summary statistics overall and for each site, which can be informally compared with the degree to which it is thought each site was able to fully implement the intervention. Variation over time within each site will also be explored, and the proportions will be compared between the rapid sequencing and delayed sequencing intervention periods.

The main analyses for the primary and secondary outcomes will be carried out on an intention-to-treat (ITT) basis according to the defined study phases. However, sensitivity analyses will be conducted excluding study sites and/or periods with suboptimal implementation of the study intervention, both in terms of overall population sequencing coverage for HOCIs and the turnaround time for sequence reports being returned to IPC teams.

For outcomes defined in both the baseline and intervention periods, such as incidence of IPC-defined HAIs and the number IPC-defined hospital outbreaks this can be informally compared

For peer review only - http://bmjopen.bmj.com/site/about/guidelines.xhtml

between the baseline, intervention and (where implementation is justified) final control periods within sites. A formal analysis will be conducted based on negative binomial regression to detect the change in the incidence rate of each event type between baseline, intervention and control phases within site, including the current proportion of inpatients who are SARS-CoV-2 positive as a fixed effect and exposure 'determined' by the number of SARS-CoV-2 negative inpatients in that week. These analyses will also include adjustment for the proportion of HCWs at each site who have received at least one vaccine dose, and a smoothed adjustment for calendar time. This will lead to an adjusted incidence rate ratio for the intervention effect, presented with a 95% confidence interval.

Missing data will be identified, and efforts made to obtain the data. In the event that some sites are unable to implement the intervention fully during the intervention period then analysis will be repeated excluding such sites to provide a 'per protocol' analysis.

A detailed statistical analysis plan (SAP) will be produced prior to commencement of analysis and agreed by the joint Trial Steering Committee and Data Monitoring Committee (TSC-DMC). All statistical tests will use a 2-sided p-value of 0.05, unless otherwise specified. All statistical analysis will be performed using Stata (StataCorp, College Station, TX, USA).

HEALTH ECONOMIC ANALYSIS

We will examine whether rapid SARS-CoV-2 genomic sequencing might lead to measurable economic advantages. A cost-benefit analysis will be conducted looking at the incremental cost or savings for the two sequencing approaches against baseline in the group of sites influenced by the time to sequence data result.

The cost of SARS-CoV-2 genome sequencing, generating the report and additional resources involved in teams training and review of the report will be obtained from the participating laboratories and study sites.

HOCI resource use will be obtained from hospital records and CRFs supplemented with information obtained from members of the IPC teams at each site to inform IPC action related cost. Costs will be evaluated from the NHS setting perspective over the study period. Economic benefits include the attributable cost savings from reducing the delay to initiate IPC measures to avert infection transmission, an estimate of the hospital cost savings due to excess bed days, and days off work by HCWs.

For peer review only

For peer review only - http://bmjopen.bmj.com/site/about/guidelines.xhtml

Mean costs and standard deviations for all phases of the study will be calculated. We will estimate the incremental mean difference in total costs between intervention phases and baseline of the study and 95% confidence intervals.

Deterministic sensitivity analysis will be performed to assess the impact of varying resource use and other relevant parameters to identify variables with the highest impact on costs.

Adjustments will be made for variation in HOCI levels due to impact of B.1.1.7 variant of SARS-CoV-2 in the UK, as well as the national COVID-19 vaccine rollout.

A Health Economic Analysis Plan will be prepared for the study prior to commencing data analysis and will be approved by the TSC-DMC.

PROCESS EVALUATION

Process evaluations are now considered integral to understanding the factors which shape outcomes achieved within a study, enrich interpretation of findings and facilitate better understandings of how the intervention may be used in other settings to create sustainable health change.

The process evaluation embedded within the HOCI study aims to understand how the rapid genome sequencing intervention works in practice across different sites.

The team will first develop initial programme theory for the SRT in advance of implementation. Programme theory describes the salient parts of the context in which the intervention will be implemented, the specific nature of the problem being addressed, the content or components of the intervention, the mechanisms through which the intervention works and how the intervention led to expected and unanticipated outcomes. Including the development of the programme theory as part of the study ensures that the team have de-risked the intervention as far as possible by anticipating and mitigating potential problems or limiting factors.[12]

Data will then be gathered using a topic guide based on the programme theory. A purposive sample of HCWs involved in the chain of activity associated with implementing the SRT across five study sites will be interviewed. Interviews will take place during or soon after the rapid phase and focus on how the SRT and HOCI study more broadly have been implemented. A structured thematic analysis of the data will be conducted using the core elements of the initial programme theory, which will then be refined and used to share learning on HOCI and facilitate transferable knowledge and sustainable future healthcare.

For peer review only - http://bmjopen.bmj.com/site/about/guidelines.xhtml

STUDY TIMELINES

Sites will be opened using a staggered approach from October 2020 to January 2021 in order to provide the greatest likelihood of each phase of site activity covering peaks, troughs and moderate incidences of community prevalence and therefore likely hospital admissions of COVID-19 patients.

Patient recruitment at sites will run for six months from late October 2020 to end April 2021.

See Table 1 for Study Schedule.

DISCUSSION

By defining and reporting SARS-CoV-2 genotype frequencies within its sites and comparing to those in the wider community, the study has the potential to overcome some of the inherent barriers to identifying the likely transmission chains. The data generated will provide an accurate as possible a picture, given the constraints of viral genetic diversity, of the number and location of SARS-CoV-2 infections acquired by nosocomial transmission, and to an extent inform how these transmissions are occurring.

While COG-UK will provide data on the utility of viral genomics for national public health planning, COG-UK HOCI will quantify the utility of the same data for local management of nosocomial infection, including whether observed benefits are time dependent and deliver the best estimates of how viral sequence data can be used to identify HAIs among HOCIs.

Study outputs will further inform decisions about the likely future use of viral genome sequencing for the management of epidemics and pandemics and how it might best be organised - centralised or diversified - to deliver maximal impact.

STUDY MONITORING

An independent joint Trial Steering Committee and Data Monitoring Committee (TSC-DMC) will be formally responsible for the oversight of the study and ensuring it is conducted in compliance with ICH Good Clinical Practice and other relevant regulations. The TSC-DMC will also advise on the need for the fourth phase of the study (a second baseline period).

For peer review only

The Trial Management Group (TMG) will be responsible for the execution of the study. Site monitoring will be undertaken by the Trial Manager, based at the UCL Comprehensive Clinical Trials Unit.

Site teams will only report adverse events which meet both the 'seriousness' threshold and are also considered 'related' to the study intervention. This was considered risk appropriate for the study as no patient-specific procedures are undertaken, and has been approved by the Ethics Committee.

PATIENT AND PUBLIC INVOLVEMENT

The COG-UK HOCI study was designed between April and May 2020 and was initially intended to run during the first wave of the COVID-19 epidemic in the UK, and therefore for timing and safety reasons, patients with COVID-19 were not directly included to participate in the study's design development.

ETHICS AND DISSEMINATION

The National Research Ethics Service Committee (Cambridge South) reviewed the study protocol (approved 23rd April 2020, REC ref 20/EE/0118). Any subsequent amendments to these documents will be submitted for further approval. The findings of this study will be disseminated through peer-reviewed publications and conference presentations.

POST-STUDY ACCESS TO DATA

The terms of the funding requires the COG-UK HOCI study dataset to be shared on UCL's Data Repository so that the anonymised individual participant data may be reused on an open policy by other researchers. This will be done within 6 months of public reporting of results and available for 5 years.

For peer review only - http://bmjopen.bmj.com/site/about/guidelines.xhtml

Table 1 - Study Schedule

Timepoint (site dependent)	6 months	4 weeks	8 weeks	4 weeks	4 weeks	6 Months
**Study stage (site dependent)**	**Set up**	**First Baseline / Control (daily)**	**Intervention sequencing result <48 hours (daily)***	**Intervention sequencing result >4 days (daily)***	**Second Baseline / Control (where justified, daily)**	**Data cleaning, analysis and reporting**
Site identification	X					
Site team discussion on sampling ability, staffing availability, and logistics	X					
Initiation of contracts, R&D approvals	X					
NHS samples begin to be processed locally under COG-UK approvals (not study-related)	X					
NHS samples to be processed under COG-UK approvals, either locally or at Sanger		X	X	X	X	
Intervention reports generated			X	X		
Intervention reports returned to site ICTs (<48h)			X			
Intervention reports returned to site ICTs (>4 days)				X		
ICTs evaluate reports, seeking Expert Sequence Interpretation Team views if needed			X	X		
Case reports for HOCIs		X	X	X	X	
Process evaluation - qualitative interviews /analysis			X	X	X	X
Process evaluation - programme theory development and refinement	X		X	X	X	X
Interim analysis and views from TSC-DMC whether second baseline/control state acceptable					X [A]	
Data cleaning		X	X	X	X	X
Final data lock and analysis						X
Reporting/publication						X

* The order of 'rapid' phase (<48 hour turnaround time) and 'slow' phase may be swapped prior to commencement of either upon agreement with the Sponsor. The option is offered to facilitate logistics/set-up at sites.
[A] TSC-DMC review should take place to determine whether it would be considered ethical to request sites have a second period of baseline/control (where sequencing data is not provided to IPC teams). This would only be on the basis that it is unclear from the initial baseline and intervention comparison whether there is a significant benefit; in cases where it is clear there is either benefit to the intervention or no benefit, then the second baseline would not take place.

For peer review only

Figure 1 – Example of HOCI Sequence Reporting Tool (SRT) Report Output

[Figure 1 – SRT Report.tif image about here]

For peer review only

ACKNOWLEDGEMENTS

Our thanks to the Principal Investigators: Dr Anu Chawla (Liverpool University Hospitals NHS Foundation Trust); Dr Maria-Teresa Cutino-Moguel (Barts Health NHS Trust); Prof Alison Holmes (Imperial College Healthcare NHS Trust); Dr Nick Machin (Manchester University NHS Foundation Trust); Dr Nikunj Mahida (Nottingham University Hospitals NHS Trust); Dr Taibtha Mahungu (Royal Free NHS Foundation Trust); Dr Gaia Nebbia (Guy's and St Thomas' NHS Foundation Trust); Dr Cassie Pope (St George's University Hospitals NHS Foundation Trust); Dr Kordo Saeed (University Hospital Southampton NHS Foundation Trust); Dr Tranprit Saluja (Sandwell and West Birmingham NHS Trust); Dr Gee Yen Shin (University College London Hospitals NHS Foundation Trust); Dr Thushan de Silva (Sheffield Teaching Hospitals NHS Foundation Trust); Dr Yusri Taha (Newcastle Hospitals NHS Foundation Trust); Prof Emma Thomson (NHS Glasgow and Greater Clyde) and teams for their time and support.

We also acknowledge the support of the independent members of the Joint Trial Steering Committee and Data Monitoring Committee (TSC-DMC):

Prof Marion Koopmans (Erasmus MC)

Prof Walter Zingg (University of Geneva)

Prof Colm Bergin (Trinity College Dublin)

Prof Karla Hemming (University of Birmingham)

Prof Katherine Fielding (LSHTM)

As well as TSC-DMC non-independent members:

Prof Nick Lemoine (NIHR CRN)

Prof Sharon Peacock (COG-UK)

We would also thank members of COG-UK who have directly supported the study:

Dr Ewan Harrison (Cambridge University)

Dr Katerina Galai (PHE)

Dr Francesc Coll (LSHTM)

Dr Michael Chapman (HDR-UK)

Prof Thomas Connor and team (Cardiff University)

Prof Nick Loman and team (University of Birmingham)

For peer review only

For peer review only - http://bmjopen.bmj.com/site/about/guidelines.xhtml

We also thank the COG-UK Consortium, and the UK National Institute for Health Research Clinical Research Network (NIHR CRN).

COLLABORATORS

MRC–University of Glasgow Centre for Virus Research (CVR), Glasgow, UK

Dr Joshua Singer, Dr Joseph Hughes and Prof Emma Thomson in supporting design and implementation of the HOCI sequence reporting tool.

University of Sheffield, Sheffield, UK

Dr Matthew Parker supporting design and testing of the HOCI sequence reporting tool.

UCL, London, UK

Dr Asif Tamuri, Dr Sunando Roy and Dr Stefan Piatek supporting design, implementation and testing of the HOCI sequence reporting tool.

CONTRIBUTORS

JBl developed the study protocol and contributed to the writing of the manuscript. OS and AC were involved in development of the statistical analysis facets of the trial and contributed to the writing of the manuscript. MP was involved in development of the health economic analysis facets of the study and contributed to the writing of the manuscript. PF and FM were involved in development of the process evaluation of the study and contributed to the writing of the manuscript. LH is the trial manager for the study. JP, PD, CP, TS, GN and LS are responsible for the operationalisation of study procedures. RM will contribute to data collection and management. JBr is the chief investigator for the study. All authors critically reviewed and approved the final version.

FUNDING

The COG-UK HOCI study is funded by the COG-UK Consortium, which is supported by funding from the Medical Research Council (MRC) part of UK Research & Innovation (UKRI), the National Institute of Health Research (NIHR) and Genome Research Limited, operating as the Wellcome Sanger Institute (UKRI MRC Grant number MC PC 4050788737).

SPONSOR

The COG-UK HOCI study is sponsored by University College London, represented by the Comprehensive Clinical Trials Unit (UCL CCTU).

COMPETING INTERESTS

None declared.

For peer review only

REFERENCES

1. Abbas M, Nunes T, Martischang R et al. Nosocomial transmission and outbreaks of coronavirus disease 2019: the need to protect both patients and healthcare workers. *Antimicrob Resist Infect Control* 2021; 10(7) doi: 10.1186/s13756-020-00875-7
2. Roy S, Hartley J, Dunn H et al. Whole-genome Sequencing Provides Data for Stratifying Infection Prevention and Control Management of Nosocomial Influenza A. *Clin Infect Dis* 2019; 69, 1649-1656, doi:10.1093/cid/ciz020.
3. Houldcroft C, Roy S, Morfopoulou S et al. Use of Whole-Genome Sequencing of Adenovirus in Immunocompromised Pediatric Patients to Identify Nosocomial Transmission and Mixed-Genotype Infection. *J Infect Dis* 2018; 218, 1261-1271, doi:10.1093/infdis/jiy323
4. Brown J, Roy S, Shah D et al. Norovirus Transmission Dynamics in a Pediatric Hospital Using Full Genome Sequences. *Clin Infect Dis* 2019; 68, 222-228, doi:10.1093/cid/ciy438
5. Meredith L, Hamilton W, Warne B et al. Rapid implementation of SARS-CoV-2 sequencing to investigate cases of health-care associated COVID-19: a prospective genomic surveillance study. *Lancet Infect Dis* 2020; 20(11), 1263-1271, doi: 10.1016/S1473-3099(20)30562-4
6. Moore G, Rickard H, Stevenson D et al. Detection of SARS-CoV-2 within the healthcare environment: a multi-centre study conducted during the first wave of the COVID-19 outbreak in England. *J Hosp Inf* 2021; 108, 189-196, doi: 10.1016/j.jhin.2020.11.024
7. Page A, Mather A, Le-Viet T et al. Large-scale sequencing of SARS-CoV-2 genomes from one region allows detailed epidemiology and enables local outbreak management. *Microbial Genomics* 2021; 7(6) doi: 10.1099/mgen.0.000589
8. Bedford, T. Phylodynamic estimation of incidence and prevalence of novel 362 coronavirus (nCoV) infections through time. *Virological*, accessed on 27/02/2020. http://virological.org/t/phylodynamic-estimation-of-incidence-and-prevalence-of-novel364 coronavirus-ncov-infections-through-time/391
9. COVID-19 Genomics UK (COG-UK) consortium. An integrated national scale SARS-CoV-2 genomic surveillance network. *Lancet Microbe* 1(3), 2020; 99-100, https://doi.org/10.1016/S2666-5247(20)30054-9
10. Stirrup O, Hughes J, Parker M et al. Rapid feedback on hospital onset SARS-CoV-2 infections combining epidemiological and sequencing data. *eLife* 2021; 10:e65828 doi: 10.7554/eLife.65828
11. Flowers P, Mapp F, Blackstone J et al. Developing Initial Programme Theory: COVID-19 Genomics UK Consortium Hospital-onset COVID-19 Study (COG-UK HOCI). SocArXiv. Accessed on 10/02/2021. doi:10.31235/osf.io/ysm35.
12. Public Health England. COVID-19: epidemiological definitions of outbreaks and clusters. Gov.uk, accessed on 03/04/2021. https://www.gov.uk/government/publications/covid-19-epidemiological-definitions-of-outbreaks-and-clusters/covid-19-epidemiological-definitions-of-outbreaks-and-clusters-in-particular-settings

For peer review only - http://bmjopen.bmj.com/site/about/guidelines.xhtml

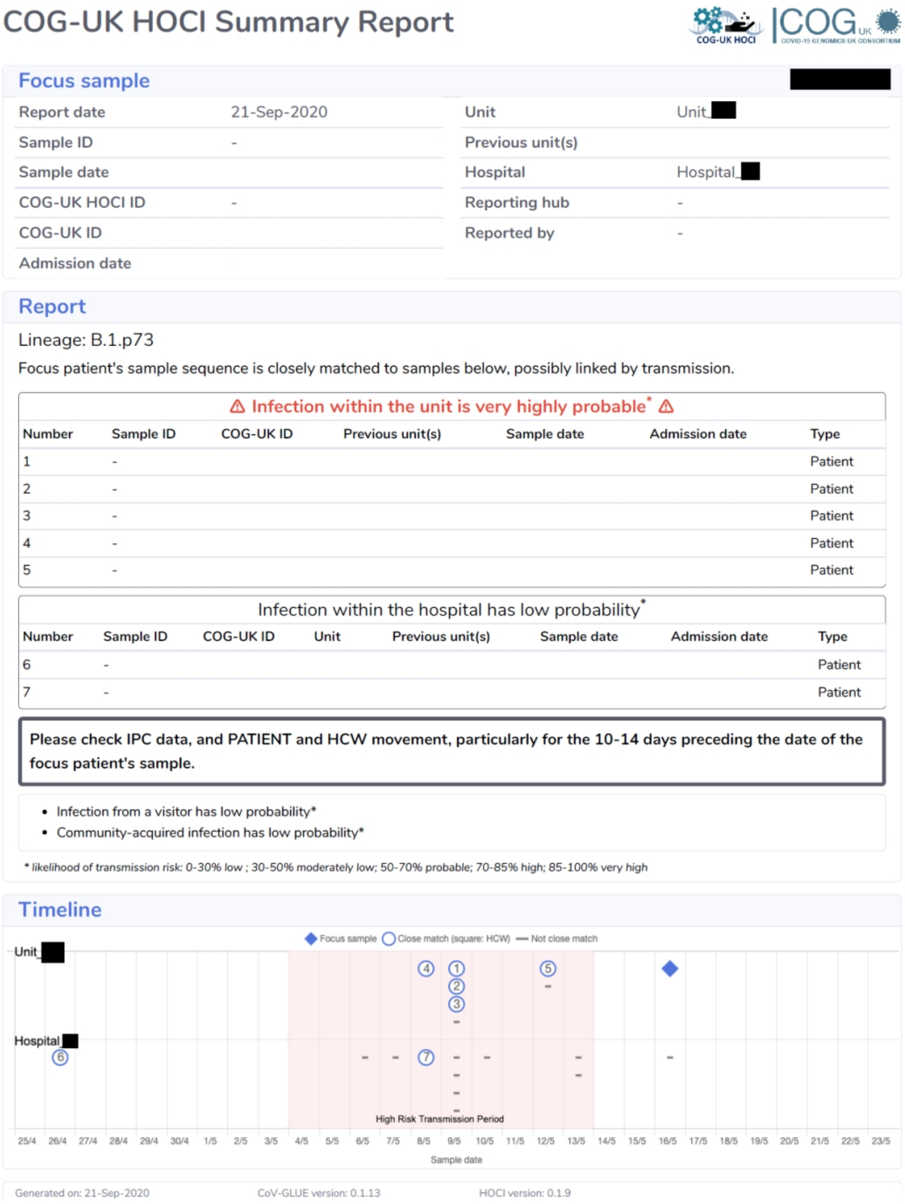
COG-UK HOCI Summary Report

Focus sample [redacted]

Report date	21-Sep-2020	Unit	Unit_[redacted]
Sample ID	-	Previous unit(s)	
Sample date		Hospital	Hospital_[redacted]
COG-UK HOCI ID	-	Reporting hub	-
COG-UK ID		Reported by	-
Admission date			

Report

Lineage: B.1.p73

Focus patient's sample sequence is closely matched to samples below, possibly linked by transmission.

⚠ Infection within the unit is very highly probable* ⚠

Number	Sample ID	COG-UK ID	Previous unit(s)	Sample date	Admission date	Type
1	-					Patient
2	-					Patient
3	-					Patient
4	-					Patient
5	-					Patient

Infection within the hospital has low probability*

Number	Sample ID	COG-UK ID	Unit	Previous unit(s)	Sample date	Admission date	Type
6	-						Patient
7	-						Patient

Please check IPC data, and PATIENT and HCW movement, particularly for the 10-14 days preceding the date of the focus patient's sample.

- Infection from a visitor has low probability*
- Community-acquired infection has low probability*

** likelihood of transmission risk: 0-30% low ; 30-50% moderately low; 50-70% probable; 70-85% high; 85-100% very high*

Timeline

Generated on: 21-Sep-2020 CoV-GLUE version: 0.1.13 HOCI version: 0.1.9

Figure 1. Sequence Reporting Tool (SRT) example

SPIRIT

STANDARD PROTOCOL ITEMS: RECOMMENDATIONS FOR INTERVENTIONAL TRIALS

SPIRIT 2013 Checklist: Recommended items to address in a clinical trial protocol and related documents*

Section/item	Item No	Description	Addressed on page number
**Administrative information**			
Title	1	Descriptive title identifying the study design, population, interventions, and, if applicable, trial acronym	1
Trial registration	2a	Trial identifier and registry name. If not yet registered, name of intended registry	3
	2b	All items from the World Health Organization Trial Registration Data Set	3
Protocol version	3	Date and version identifier	2
Funding	4	Sources and types of financial, material, and other support	20
Roles and responsibilities	5a	Names, affiliations, and roles of protocol contributors	20
	5b	Name and contact information for the trial sponsor	20
	5c	Role of study sponsor and funders, if any, in study design; collection, management, analysis, and interpretation of data; writing of the report; and the decision to submit the report for publication, including whether they will have ultimate authority over any of these activities	20
	5d	Composition, roles, and responsibilities of the coordinating centre, steering committee, endpoint adjudication committee, data management team, and other individuals or groups overseeing the trial, if applicable (see Item 21a for data monitoring committee)	19
**Introduction**			
Background and rationale	6a	Description of research question and justification for undertaking the trial, including summary of relevant studies (published and unpublished) examining benefits and harms for each intervention	4
	6b	Explanation for choice of comparators	5
Objectives	7	Specific objectives or hypotheses	5

For peer review only - http://bmjopen.bmj.com/site/about/guidelines.xhtml

Trial design	8	Description of trial design including type of trial (eg, parallel group, crossover, factorial, single group), allocation ratio, and framework (eg, superiority, equivalence, noninferiority, exploratory)	5

Methods: Participants, interventions, and outcomes

Study setting	9	Description of study settings (eg, community clinic, academic hospital) and list of countries where data will be collected. Reference to where list of study sites can be obtained	8
Eligibility criteria	10	Inclusion and exclusion criteria for participants. If applicable, eligibility criteria for study centres and individuals who will perform the interventions (eg, surgeons, psychotherapists)	8
Interventions	11a	Interventions for each group with sufficient detail to allow replication, including how and when they will be administered	6
	11b	Criteria for discontinuing or modifying allocated interventions for a given trial participant (eg, drug dose change in response to harms, participant request, or improving/worsening disease)	N/A
	11c	Strategies to improve adherence to intervention protocols, and any procedures for monitoring adherence (eg, drug tablet return, laboratory tests)	N/A
	11d	Relevant concomitant care and interventions that are permitted or prohibited during the trial	N/A
Outcomes	12	Primary, secondary, and other outcomes, including the specific measurement variable (eg, systolic blood pressure), analysis metric (eg, change from baseline, final value, time to event), method of aggregation (eg, median, proportion), and time point for each outcome. Explanation of the clinical relevance of chosen efficacy and harm outcomes is strongly recommended	9-10
Participant timeline	13	Time schedule of enrolment, interventions (including any run-ins and washouts), assessments, and visits for participants. A schematic diagram is highly recommended (see Figure)	17
Sample size	14	Estimated number of participants needed to achieve study objectives and how it was determined, including clinical and statistical assumptions supporting any sample size calculations	10-11
Recruitment	15	Strategies for achieving adequate participant enrolment to reach target sample size	8

Methods: Assignment of interventions (for controlled trials)

Allocation:			N/A

For peer review only - http://bmjopen.bmj.com/site/about/guidelines.xhtml

Sequence generation	16a	Method of generating the allocation sequence (eg, computer-generated random numbers), and list of any factors for stratification. To reduce predictability of a random sequence, details of any planned restriction (eg, blocking) should be provided in a separate document that is unavailable to those who enrol participants or assign interventions	
Allocation concealment mechanism	16b	Mechanism of implementing the allocation sequence (eg, central telephone; sequentially numbered, opaque, sealed envelopes), describing any steps to conceal the sequence until interventions are assigned	N/A
Implementation	16c	Who will generate the allocation sequence, who will enrol participants, and who will assign participants to interventions	N/A
Blinding (masking)	17a	Who will be blinded after assignment to interventions (eg, trial participants, care providers, outcome assessors, data analysts), and how	N/A
	17b	If blinded, circumstances under which unblinding is permissible, and procedure for revealing a participant's allocated intervention during the trial	N/A

Methods: Data collection, management, and analysis

Data collection methods	18a	Plans for assessment and collection of outcome, baseline, and other trial data, including any related processes to promote data quality (eg, duplicate measurements, training of assessors) and a description of study instruments (eg, questionnaires, laboratory tests) along with their reliability and validity, if known. Reference to where data collection forms can be found, if not in the protocol	11
	18b	Plans to promote participant retention and complete follow-up, including list of any outcome data to be collected for participants who discontinue or deviate from intervention protocols	N/A
Data management	19	Plans for data entry, coding, security, and storage, including any related processes to promote data quality (eg, double data entry; range checks for data values). Reference to where details of data management procedures can be found, if not in the protocol	11
Statistical methods	20a	Statistical methods for analysing primary and secondary outcomes. Reference to where other details of the statistical analysis plan can be found, if not in the protocol	12
	20b	Methods for any additional analyses (eg, subgroup and adjusted analyses)	12-13
	20c	Definition of analysis population relating to protocol non-adherence (eg, as randomised analysis), and any statistical methods to handle missing data (eg, multiple imputation)	12

Methods: Monitoring

For peer review only - http://bmjopen.bmj.com/site/about/guidelines.xhtml

Data monitoring	21a	Composition of data monitoring committee (DMC); summary of its role and reporting structure; statement of whether it is independent from the sponsor and competing interests; and reference to where further details about its charter can be found, if not in the protocol. Alternatively, an explanation of why a DMC is not needed	19
	21b	Description of any interim analyses and stopping guidelines, including who will have access to these interim results and make the final decision to terminate the trial	N/A
Harms	22	Plans for collecting, assessing, reporting, and managing solicited and spontaneously reported adverse events and other unintended effects of trial interventions or trial conduct	15
Auditing	23	Frequency and procedures for auditing trial conduct, if any, and whether the process will be independent from investigators and the sponsor	N/A
**Ethics and dissemination**			
Research ethics approval	24	Plans for seeking research ethics committee/institutional review board (REC/IRB) approval	16
Protocol amendments	25	Plans for communicating important protocol modifications (eg, changes to eligibility criteria, outcomes, analyses) to relevant parties (eg, investigators, REC/IRBs, trial participants, trial registries, journals, regulators)	N/A
Consent or assent	26a	Who will obtain informed consent or assent from potential trial participants or authorised surrogates, and how (see Item 32)	8-9
	26b	Additional consent provisions for collection and use of participant data and biological specimens in ancillary studies, if applicable	N/A
Confidentiality	27	How personal information about potential and enrolled participants will be collected, shared, and maintained in order to protect confidentiality before, during, and after the trial	9
Declaration of interests	28	Financial and other competing interests for principal investigators for the overall trial and each study site	20
Access to data	29	Statement of who will have access to the final trial dataset, and disclosure of contractual agreements that limit such access for investigators	16
Ancillary and post-trial care	30	Provisions, if any, for ancillary and post-trial care, and for compensation to those who suffer harm from trial participation	N/A
Dissemination policy	31a	Plans for investigators and sponsor to communicate trial results to participants, healthcare professionals, the public, and other relevant groups (eg, via publication, reporting in results databases, or other data sharing arrangements), including any publication restrictions	16
	31b	Authorship eligibility guidelines and any intended use of professional writers	20

For peer review only

For peer review only - http://bmjopen.bmj.com/site/about/guidelines.xhtml

	31c	Plans, if any, for granting public access to the full protocol, participant-level dataset, and statistical code	N/A
**Appendices**			
Informed consent materials	32	Model consent form and other related documentation given to participants and authorised surrogates	N/A
Biological specimens	33	Plans for collection, laboratory evaluation, and storage of biological specimens for genetic or molecular analysis in the current trial and for future use in ancillary studies, if applicable	N/A

*It is strongly recommended that this checklist be read in conjunction with the SPIRIT 2013 Explanation & Elaboration for important clarification on the items. Amendments to the protocol should be tracked and dated. The SPIRIT checklist is copyrighted by the SPIRIT Group under the Creative Commons "Attribution-NonCommercial-NoDerivs 3.0 Unported" license.

For peer review only

For peer review only - http://bmjopen.bmj.com/site/about/guidelines.xhtml
